# Progress and Challenges of Predictive Biomarkers for Immune Checkpoint Blockade

**DOI:** 10.3389/fonc.2021.617335

**Published:** 2021-03-11

**Authors:** Yanna Lei, Xiaoying Li, Qian Huang, Xiufeng Zheng, Ming Liu

**Affiliations:** Department of Abdominal Oncology, West China Hospital, Sichuan University, Chengdu, China

**Keywords:** biomarkers, immune-checkpoint blockade, cancer, T cells, PD-1

## Abstract

Over the past decade, immune checkpoint blockade (ICB) therapy has revolutionized the outlook for oncology with significant and sustained improvement in the overall patient survival. Unlike traditional cancer therapies, which target the cancer cells directly, ICB acts on the immune system to enhance anti-tumoral immunity. However, the response rate is still far from satisfactory and most patients are refractory to such treatment. Unfortunately, the mechanisms underlying such heterogeneous responses between patients to ICB therapy remain unclear. In addition, escalating costs of cancer care and unnecessary immune-related adverse events also are pertinent considerations with applications of ICB. Given these issues, identifying explicit predictive biomarkers for patient selection is an urgent unmet need to increase the efficacy of ICB therapy. The markers can be classified as tumor related and non-tumor-related biomarkers. Although substantial efforts have been put into investigating various biomarkers, none of them has been found to be sufficient for effectively stratifying patients who may benefit from immunotherapy. The present write up is an attempt to review the various emerging clinically relevant biomarkers affecting the efficacy of immune checkpoint inhibitors, as well as the limitations associated with their clinical application.

## Introduction

For years, conventional oncological treatments such as surgery, chemotherapy, radiotherapy, as well as targeted therapy remained the cornerstones for cancer treatment. However, the advent of immunotherapy, especially immune checkpoint blockade (ICB) therapy, has significantly changed the therapeutic landscape of several cancer types (1). The checkpoint molecules including cytotoxic T lymphocyte-associated protein 4 (CTLA-4) and programmed death 1 (PD-1), together with programmed death-ligand 1 (PD-L1) have been successfully targeted and antibodies affecting these molecules have been approved by the US Food and Drug Administration for therapy against several cancer types. In addition, the clinical trials of immune checkpoint inhibitors have showed remarkable anti-tumor activities. These encouraging results can drive the development of more immune checkpoint blockage molecules.

The immune system serves as the last line of defense against the formation and progression of tumors. The cancer immunity cycle, first put forward by Chen and Mellman, refers to a series of stepwise events that can be initiated and then expanded iteratively for an anticancer immune response to destroy the cancer cells (2). This cycle can be divided into three major steps: release of cancer antigens and their presentation to T cells, then T cells get activated and reach the tumor sites, and the killing of cancerous cells by cytotoxic T lymphocytes (CTLs) resulting in significant antigen release. The activation of T cells relies on two distinct signals. The first one is the binding of T cell receptor (TCR) to complexes of antigenic peptides with major histocompatibility complex (MHC). The second one is generated from the modulation of co-stimulatory or co-inhibitory molecules that could facilitate the tumor escape from immune surveillance (3). These molecules are also called immune checkpoints. ICB can disrupt the potential effects of immune checkpoints and cause an optimum activation of cytotoxic T cells to effectively kill cancer cells. Despite its success in various malignancies, only a small population of cancer patients have actually benefited from ICB. For example, Ipilimumab, the first CTLA-4 antibody approved by FDA could improve overall survival in patients with advanced melanoma in 2011 (4), was reported to be effective in <11% patients. Melanoma is one of the tumors that has exhibited a favorable response rate to immunotherapy. According to the results of KEYNOTE-006 phase III study, the overall response rate of Pembrolizumab, the first PD-1 inhibitor approved for refractory, advanced melanoma, was about 42% (5). Although ICB demonstrated significant anti-tumor effects, the objective response rates of monotherapy were far from satisfactory in the clinical trials. Moreover, the antibody drugs have substantially increased socioeconomic burdens and produced immune-related adverse events. Thus, identifying various biomarkers to distinguish patients that might respond to immunotherapy would significantly decrease treatment cost, avoid undesirable immunotoxicities and promote the development of precision medicine. Despite significant efforts to identify such biomarkers, the predictive role of different biomarkers remains unclear. It has been perceived that monitoring the change of any index, which may have value in selecting patients, will be challenging. The lack of uniform standard detection methods and analysis methods can hamper the application of these biomarkers. Additionally, a single biomarker may not be suitable to accurately predict the selection of the patient for immunotherapy.

This review summarizes recent data on the various biomarkers for predicting response of cancer patients to ICB therapy, and its relevance to various cancer biomarkers, tumor microenvironment-related biomarkers, circulating prognostic biomarkers as well as host-related biomarkers.

## Tumor-Related Biomarkers for ICB

### The Expression of PD-L1

PD-L1, one of the ligands of PD-1, has been reported to be expressed not only on tumor cells but also on other host immune cells such as dendritic cells, macrophages and T cells (6). Tumor cells can effectively escape from the immune surveillance through exploiting PD-1/PD-L1 signaling pathway. PD-L1 can act as an important target of ICB. The expression levels of PD-L1 were the most common immune-based biomarker found to be affected in current clinical practice (7–9). For instance, patients overexpressing PD-L1 are more likely to show a better prognosis and benefit from ICB (8, 10, 11). It may display predictive value in some certain tumor types (in Table 1, different tumor types have been indicated that have gained FDA approval and had a companion diagnostic PD-L1 testing in last 3 years). For example, PD-L1 level in advanced non-small-cell lung cancer (NSCLC) patients is considered as an essential predictor of patient response to pembrolizumab. According to the results of KEYNOTE-024 phase III clinical trial, if NSCLC patients bear PD-L1 expression upon at least 50% of cancer cells, pembrolizumab, rather than chemotherapy, is to be used as the first-line treatment (12). In metastatic triple-negative breast cancer patients with PD-L1–positive (expression of PD-L1 on the tumor-infiltrating immune cells ≥1% of tumor area), combination therapy with atezolizumab plus nab-paclitaxel led to a significantly longer progression free survival than placebo plus nab-paclitaxel (13).

**Table 1:** A brief update on various approvals granted by FDA for immune checkpoint inhibitors linked to PD-L1 detection in the past 3 years.

**Trial**	**Tumor type**	**Drug**	**Year of approval**	**Line of therapy**	**PD-L1 threshold**	**Companion diagnostic**	**Number of patients**	**Endpoint**	**Results**
KEYNOTE-355	TNBC	Pembrolizumab + chemotherapy VS Placebo + chemotherapy	2020	1st	CPS^c^ ≥ 10	IHC 22C3	566	PFS, OS	Median PFS: 9.7 vs. 5.6 m
IMpower110	adult patients with NSCLC	Atezolizumab VS Platinum-based chemotherapy	2020	1st	TC ≥ 50% or IC ≥ 10%	SP142	277	OS	Median OS: 20.2 vs 13.1 m Median PFS: 8.1 vs. 5.0 m
CHECKMATE-227 (Part 1a)	NSCLC	Nivolumab+ ipilimumab VS Platinum-doublet chemotherapy	2020	1st	TC ≥ 1%	IHC 28-8	396	OS	Median OS: 17.1 vs. 14.9 m
KEYNOTE-181	ESCC^a^	Pembrolizumab VS Chemotherapy	2019	2nd	CPS ≥10	IHC 22C3	85	OS	Median OS: 10.3 vs. 6.7 m
KEYNOTE-180		Pembrolizumab		3rd			121	ORR, response duration	ORR: 20%
KEYNOTE-042	NSCLC	Pembrolizumab V.S Carboplatin-containing chemotherapy	2019	1st	TPS^d^ ≥ 1%	IHC 22C3	637	OS	Median OS: TPS ≥ 1%: 16.7 vs. 12.1 m TPS ≥ 20%: 17.7 vs. 13.0 m TPS ≥ 50%: 20 vs. 12.2 m
IMpassion130	TNBC	Atezolizumab + nab-paclitaxel VS Placebo + nab-paclitaxel	2019	1st	IC ≥ 1%	SP142	451	PFS, OS	Median OS: 7.4 vs. 4.8 m
KEYNOTE-048 subgroups	HNSCC	Pembrolizumab VS Cetuximab plus chemotherapy	2019		CPS ≥ 1	IHC 22C3	301	OS	Median OS: CPS ≥1: 12.3 vs. 10.3 m CPS ≥ 20: 14.9 vs. 10.7 m
KEYNOTE-158	Cervical cancer	Pembrolizumab	2018	2nd	CPS ≥ 1	IHC 22C3	98	ORR	ORR:14.3%^b^
KEYNOTE-059	Gastric/GEJ	Pembrolizumab	2017	3rd	CPS ≥ 1	IHC 22C3	259	ORR	Overall ORR: 11.6% PD-L1+:15.5% PD-L1-: 6.4%
NCT01693562	Urothelial carcinoma	Durvalumab	2017	2nd	TC or IC≥ 25%	SP263	191	ORR	Overall ORR: 17.8% PD-L1+:27.6% PD-L1-: 5.1%

a *Efficacy of pembrolizumab was investigated in two clinical trials, KEYNOTE-181 and KEYNOTE-180*.

b *ORR was 14.3% with a median follow-up time of 11.7 months in 77 patients with PD-L1–positive tumors. No responses were observed in patients whose tumors did not have PD-L1 expression*.

c *TPS is calculated as the ratio between the number of PD-L1+ tumor cells and the total number of tumor cells*.

d *CPS is calculated as the ratio between the number of all PD-L1+ cells and the total number of cells*.

*TNBC, triple-negative breast cancer; CPS, combined positive score; NSCLC, non-small cell lung cancer; GEJ, gastroesophageal junction; IC, immune cells; TC, tumor cells; TPS, tumor proportion score; HNSCC, head and neck squamous cell carcinoma; ESCC, squamous cell carcinoma of the esophagus*.

However, the imperfect nature of PD-L1 as a biomarker has been reported in some studies and a subset of patients with high PD-L1 expression do not benefit from ICB. For example, in patients with advanced HCC (Hepatocellular Carcinoma), the predictive value of PD-L1 expression on tumor cells did not directly correlate with the patient response to anti-PD1 therapy according to the data from CheckMate 040 clinical trial (14). In this trial, the response rate of the patients with PD-L1 expression on at least 1% of cancer cells was 26% and on <1% of cancer cells was 19%. The study CheckMate-032 also revealed that PD-L1 status could not serve as an effective biomarker in patients with urothelial cancer (15). Davis et al. evaluated the predictive role of PD-L1 based in pivotal clinical trials which led to FDA approval of immune checkpoint inhibitors between 2011 and 2019 (16). They concluded that PD-L1 was predictive in only 28.9% of cases and only 20% of those reported displayed companion PD-L1 diagnostic testing. They also pointed out different types of tissues that were tested (fresh vs. archival), PD-L1 expression cutoffs as well as type of cells test for the PD-L1 expression that could explain the heterogeneity in PD-L1 predictiveness. In certain cancer patients, although high PD-L1 expression was detected, tumor-infiltrating T cells were found to be relatively scarce, which may explain patients' lack of response to ICB (17). These findings indicated that PD-L1 status should not be considered as the sole predictive biomarker to determine whether ICB can be used clinically.

Additionally, PD-L1 expression on the immune cells can possibly predict an increased response to anti-PD1/PD-L1 treatment in metastatic bladder cancer and breast cancer (18). Furthermore, Kleinovink et al. reported previously that PD-L1 blockade was still effective against PD-L1 knockout tumors in mice and PD-L1 expression on immune cells was not substantially altered by the lack of PD-L1 expression on the cancer cells. They demonstrated that PD-L1 expression on tumor cells was not a prerequisite for the effectiveness of anti-PD-L1 therapy. Instead, its expression on immune cells may be more predictive of possible therapeutic effects of ICB (19). However, these results were based on the findings for only certain cancer types and need more evidence-based research.

The application of PD-L1 expression as a predictive marker can be also affected by other issues. For example, intratumoral heterogeneity may have a significant impact on the data interpretation of the results gained from analysis of a single-region of a tumor sample (20). Moreover, there is no established universal standard available about the cut-off points for PD-L1 expression (21). PD-L1 expression is currently quantified by immunohistochemistry, but the PD-L1 staining can be heterogeneous given the availability of multiple staining antibodies and interpretation protocols. SP142 (Ventana Medical Systems), SP263 (Ventana Medical Systems), and IHC 22C3 (Dako North America, Inc.) are the common antibodies that have been used in clinical practice. These drawbacks may explain why a fraction of PD-L1-positive tumors do not effectively respond to ICB, whereas PD-L1-negative tumors can respond.

Moreover, as overexpression of PD-L1 in HCC tumors generally indicated a poor prognosis and vascular formation (22), the predictive value of PD-L1 expression is controversial and PD-L1 expression as a biomarker may have limited applications.

### Tumor Mutational Burden

The efficacy of ICB therapy is determined predominantly by T cells. It has been reported that the signatures of T cell dysfunction and exclusion correlated with the clinical response to ICB (23, 24). T cell activation may be triggered by their recognition of tumor-related antigens that can be affected by tumor mutation burden (TMB). TMB is referred to the total number of non-synonymous mutations observed per megabase (25). Notably, not all the mutations can induce the production of neoantigens. Chan et al. explained the mechanism that only a minority of antigenic peptides derived from mutations can be loaded to major histocompatibility complex (MHC) molecules and ultimately recognized by T cells (26). The results suggested that the occurrence of more somatic mutations can lead to an increased neoantigen production and trigger a stronger immune response to eradicate tumor (27, 28), and thus TMB may be predictive of responsiveness to ICB therapy (29). Indeed, in several retrospective studies, the response rates to ICB for cancers with high mutational loads, such as NSCLC (30), melanoma (27), small-cell lung cancer (31), and urothelial cancer (32, 33), were found to be >15%. In general, patients with higher TMB are more likely to demonstrate an improved objective response rate and benefit from ICB. However, as tumor grows in size, they may acquire neoantigen intratumor heterogeneity (ITH) which can ultimately impact anti-tumor immunity and PD-L1 expression. Wolf et al. demonstrated intra-tumor heterogeneity can diminish immune response in melanoma and emphasized the importance of clonal mutations in immune surveillance (34). Patients bearing low neoantigen ITH and high clonal neoantigen burden can display high PD-L1 expression and significantly benefit from PD-1 blockade, with extended progression-free survival (35).

However, for some tumor types, TMB does not directly correlate with ICB efficacy. For example, Merkel-cell carcinoma (36) and renal cell carcinoma (37) show intermediate levels of TMB but still have a high response rate to ICB therapy. Merkel-cell carcinoma (MCC) is a rare neuroendocrine malignancy of the skin. The Merkel Cell Polyomavirus (MCPyV), as well as ultraviolet light radiation are the common etiological agent responsible for this malignancy. The UV mutational signature has been reported to be positively correlated with TMB-high classification. The MCPyV genomic DNA sequences were not found in any TMB-high cases but were identified in 63% of TMB-low cases. However, no significant difference in the response rate was observed between TMB-high/UV driven group and TMB-low/MCPyV-positive group (38). This discrepancy may be explained by the superior quality of neoantigens generated by intermediate levels of TMB (26). TMB also may not display a predictive value in NSCLC patients who were administered chemotherapy plus ICB or chemotherapy alone as the first-line treatment (39). In this study, although chemotherapy could increase TMB, the mutations were not observed to be immunogenic to trigger anti-tumor immune responses. Similarly, high TMB caused by radiation or cytotoxic drug exposure has been found not to directly correlate with an efficient anti-tumor response (35).

Taken together, TMB acting as an exclusive biomarker to differentiate responders from non-responders may not be sufficiently robust. However, there is no definitive threshold to define high or low TMB (40). Hence, standardization of TMB calculation is essential before practicing the clinical implementation of TMB. Although next-generation sequencing (NGS) techniques have made TMB assessment more accessible, the associated high-cost and time-consuming nature of this technique have significantly restricted its utility. Several trials that accessed TMB values were retrospective studies that primarily examined the relationship between TMB values and progression-free survival (PFS) or objective response rate (ORR) rather than overall survival (OS) only (41). Thus, several steps must be implemented carefully before TMB can serve as a possibly reliable biomarker and establish suitable TMB cutoffs is the most critical step.

### Microsatellite Instability and Mismatch Repair Deficiency

Mismatch repair pathways identify and repair mismatched bases during DNA replication and gene recombination. However, defective DNA mismatch repair (MMR) may cause uncorrected DNA replication and produce numerous aberrant neoantigens. It has been reported that MMR tumors may carry about 10 to 100 times more mutations than mismatch repair–proficient tumors (42). Also, cancers with MMR are more likely to be influenced by the repetitive DNA sequences' mutations and thus lead to a high level of microsatellite instability (MSI-H) (43). MSI was the first biomarker approved by the FDA to predict an optimal clinical response to anti-PD1 blockage regardless of tumor site or histology (44).

In 2017, pembrolizumab gained accelerated approval by the FDA to treat unresectable solid tumors with MSI-H or MMR-D (45). This approval was based on the results from 149 patients with MSI-H or MMR-D solid tumors who were enrolled in five clinical trials. In these trials, 90 patients had colorectal cancer and 59 patients had other types of cancers. The response rate was similar in colorectal cancer and non-colorectal cancer. The overall response rate was 39.6 and 78% of the response sustaining longer than 6 months. Based on the results of CheckMate 142, nivolumab was approved by the FDA for mCRC patients with MSI-H or MMR-D who had disease progression after chemotherapy (46). The results from the Phase II KEYNOTE-158 study (47) and arm Z1D—A sub-protocol of the NCI-MATCH (EAY131) study (48) also revealed that the PD-1 blockade has a promising activity for MSI-H/dMMR cancer regardless of the tumor type.

Interestingly, a favorable response of patients with MSI-H or MMR-D to ICB may be attributed to a positive correlation reported between MSI-H/MMR-D and PD-L1 expression. However, Mandal et al. found that although PD-L1 expression in MMR-D/MSI-H tumor was significantly higher than that in tumor with mismatch repair proficient, still half of the patients with MMR-D failed to respond effectively to the ICB therapy (49). They reported that MSI intensity, a parameter that could precisely quantify genomic MSI level, and mutational load, especially insertion-deletion mutational load, could explain that why a subset of patients with MMR-D did not respond to ICB. In addition, alterations in tumor antigen presenting machinery and tumor-extrinsic factors such as inadequate T-cell activation may also have an impact on the response to ICB (50).

Despite encouraging findings indicating that MSI status could serve as a potential marker to stratify responders from the non-responders, a number of ICB sensitive patients cannot be unambiguously distinguished based on MSI level. As reported with other biomarkers, MSI status also has its limitations and other biomarkers should be used in conjunction to identify the sensitive patients.

### Gene Expression Profiling

Gene expression profiling (GEP) of tumor cells could also serve as a predictive biomarker to monitor the efficacy of ICB. Hence, exploring the possible links between genomic subtypes and clinical prognosis can contribute to accurately predict the subset of patients that may respond to ICB treatment (51). Additionally, immune gene signatures such as IFN-γ signaling and T-cell–inflamed gene expression signature are related to immunotherapy response in several cancers (21). For instance, IFN-γ signaling plays a vital role in immune surveillance. CD8^+^ T cells can inhibit tumor cell proliferation and enhance the immune response by secreting IFN-γ. However, IFN-γ could also upregulate PD-L1 expression on the tumor cells, which can efficiently protect tumor cells from immune surveillance (52). Furthermore, constant exposure to IFN-γ may protect tumor cells from being eliminated by the immune system. This is primarily because tumor cells without optimal IFN-γ signaling activation might display an enhanced survival advantage (53). Ayers et al. revealed that the IFN-γ-related gene profile obtained from baseline tumor tissues was related to an overall better response and PFS in the patients with melanoma, head and neck cancer, and gastric cancer who were treated with pembrolizumab (54). In NSCLC, patients with effector T cell-associated and interferon-γ-associated gene signatures displayed an improved overall survival, upon treatment with atezolizumab. In addition, PD-L1 expression also can directly correlate with effector T cell-associated and interferon-γ-associated gene signatures (55). Moreover, in gastric cancer, T-cell–inflamed gene expression can correlate with clinical benefit in pembrolizumab-treated patients and thus T-cell–inflamed gene expression has an enormous potential to serve as a potential biomarker for patient selection (56).

Liu et al. demonstrated that the genomic and transcriptomic features could also predict the response to anti-PD-1 therapy in melanoma (24). They revealed that MHC-II expression, tumor heterogeneity, purity and ploidy correlated with ICB response. A low expression of MHC-II and high LDH was associated with increased resistance to ipilimumab (an anti-CTLA4 antibody). However, patients with lymph node metastasis were more likely to benefit from ipilimumab. They also suggested that the melanoma subtype may confound the association between TMB and response to PD-1 blockade. When stratified by melanoma subtype, the TMB may not serve as a good predictor of patient response. They also developed predictive models integrating clinical, genomic and transcriptomic characteristics and these models could accurately separate patients by PFS and OS. Notably, previous treatment of anti-CTLA-4 antibody may also affect the predictors of response to anti-PD1 therapy.

Several mutations have also been associated with the efficacy of ICB. Gainor et al. demonstrated that NSCLC patients with epidermal growth factor receptor (*EGFR*) or anaplastic lymphoma kinase (*ALK*) mutation exhibit a lower response rate to ICB treatment (57). However, NSCLC patients with *TP53* or *KRAS* mutations, especially co-occurring *TP53/KRAS* mutations, show a favorable clinical benefit to anti–PD-1 treatment (58). The study also revealed that it was *TP53* mutation but not *KRAS* mutations that could significantly enhance PD-L1 expression. *TP53* mutation can enhance CD8^+^ T cell infiltration and activate effector T cells as well as IFN-γ-associated gene signatures. Tumors with TP53 and KRAS mutations may display a higher mutation burden.

KRAS is one of the most common oncogenic drivers in lung adenocarcinoma (LUAC) and a great molecular diversity as well as phenotypic heterogeneity has been found in KRAS-mutant LUACs which could be interpreted by the co-occurring genetic events (59). STK11/LKB1 (KL), TP53 (KP), and CDKN2A/B inactivation coupled with low expression of the NKX2-1 (TTF1) transcription factor (KC) co-mutations can define distinct subgroups of KRAS-mutant LUAC. KL tumors that correlated with cold tumor immune microenvironment. STK11/LKB1 has been associated with PD-L1 expression, immune escape and innate resistance to PD-1 blockade in KRAS-mutant LUAC (60). In contrast, KP tumors were found to demonstrate higher levels of somatic mutations, infiltration of immune cells, immune checkpoint effector molecules including PD-L1, PD-1 as well as CTLA-4 and longer relapse-free survival. Hence, KP tumors were more likely to respond to PD-1 axis blockade. PD-1 expression levels on TILs also holds enormous predictive potential in the non-small-cell lung cancer patients who were treated with anti-PD-1 therapy (61). The transcriptional profile of TIL can vary widely due to the different expression levels of PD-1. CD8+ T lymphocytes with higher PD-1 expression (PD-1^T^ TILs) have increased expression of genes involved in the cell cycle regulation and proliferation. Higher expression of the proliferation marker Ki67 was also found in PD-1^T^ TIL. PD-1^T^ TIL can lose the classical CD8+ effector functions but may develop a novel effector function and thus could help in accurately predicting the efficacy of PD-1 blockade. PD-1^T^ TILs can constitutively secrete CXCL13 which could recruit other immune cell subsets to the tumor environment. Patients with tumors harboring more than 1% of PD-1^T^ cells have displayed improved OS than <1% of PD-1^T^ cells.

Polybromo-1 (PBRM1) status also has been reported to be associated with the efficacy of ICB in clear cell renal cell carcinoma (ccRCC) (62). The PBRM1 loss could impair the IFNγ signaling pathway in murine RCC cell lines and thus correlated with a less immunogenic tumor environment as well as resistance to ICB. In addition, the IMmotion150 dataset was also analyzed and it was found that patients with PBRM1 mutations had a significantly lower response rate to anti-PD-L1 therapy. Beta-2 microglobulin (B2M) mutations can also have an impact on the response to ICB in melanoma (63). B2M is a component of the MHC-I molecule and can stabilize the progress of antigens presentation. The results showed that B2M aberrations including point mutations, deletions or a loss of heterozygosity (LOH) were also closely associated with tumor evasion of CD8+ T-cell responses, disease progression and resistance to ICB. The LOH was a more common form of B2M alterations found in the non-responders.

Furthermore, *CTNNB1* status might have a potential to predict patients' response to anti-PD-1 therapy. *CTNNB1* is one of the frequently mutated genes in HCC and has been related to alcohol intake (64, 65). It has also been found to be associated with tumor classifications as well as tumor characteristics. In general, HCC with *CTNNB1* mutations show microtrabecular or pseudoglandular histological patterns, cholestatic tendencies, and T cell exclusion (65). Harding et al. implemented prospective NGS in the patients and found that CTNNB1-mutated HCC was associated with innate resistance to immune checkpoint inhibitors (66). Galarreta et al. created a novel genetically engineered mouse model and reported that CTNNB1-encoded β-catenin activation could promote immune escape and resistance to anti-PD-1 therapy (64). The mechanism was related to a defective recruitment of dendritic cells, which could in turn can impair CD8^+^ T cell infiltration. They also showed that overexpression of chemokine (C-C motif) ligand 5 (CCL5) could restore the immune surveillance in β-catenin-driven tumors. Furthermore, a recent study revealed that CTNNB1 mutation could be directly correlated with higher TMB and poor survival rate. They found that the natural killer (NK) cells positively infiltrated the CTNNB1 mutation group and they speculated that the possible reason may be due to the CD96, a novel immune checkpoint receptor in NK cells (67). Furthermore, liver cancer with *LRP1B* or *TP53* mutations have been correlated with high TMB and patients with any of these two gene mutations were more likely to respond to immunotherapy although LRP1B or TP53 mutations generally serve as poor prognostic factors (68). Recently, a notion of mutation risk score has been developed, which may help in directing personalized immunotherapy for HCC patients. More importantly, the predictive value of mutation risk score was found to be higher than TMB. The correlation between survival and mutation status of TP53, TERT, CTNNB1, BRD4, as well as MLL in patients that received immunotherapy was determined. A multivariate Cox regression analysis was used to analyze these genes. The mutation risk score was calculated as described: risk score = *TP53* × 0.0233 + *TERT* × 0.3014 + *CTNNB1* × 2.0907 + *BRD4* × 1.9596 + *MLL* × 1.0637. Patients were divided into low-risk score and high-risk score groups. PFS, as well as OS in patients with high-risk score, was reported to be significantly longer than in the patients with low-risk score (69).

The state of CD8+ T cells may also have a desired impact on the response to ICB (70). According to the genes expressed, the CD8+ T cells can be divided into two different states: CD8_G and CD8_B. CD8_G cells have an increased expression of genes related to memory, activation as well as cell survival (such as TCF7) and decreased expression of co-inhibitory molecules that are more likely to be enriched in responders. However, the CD8_B cells can display a higher expression of genes linked to cell exhaustion (e.g., CD38, LAG3, ENTPD1) and are also found to be enriched in non-responders. This study also identified that usually more responders have a higher ratio of TCF7+CD8+ T cells than the non-responders. Thus, staining of the TCF7 protein in CD8+ T cells could be a potential biomarker for predicting the efficacy of PD-1 inhibitors. For instance, ENTPD1 (CD39) can serve as a marker of the exhausted CD8+ T cells and effectively separate all TIM3+ from TIM3 cells.

In addition to exploring possible changes in genomics, identifying intracellular programs of malignant cells is equally important (71). Researchers have identified a novel malignant cell program using single-cell RNA sequencing in melanoma. The program expressed by tumor cells was found to be associated with T cell exclusion and immune evasion. Multiple immune resistance mechanisms were found to be co-regulated in this program and the program could also predict clinical responses to anti-PD-1 therapy in melanoma patients.

Taken together, these findings demonstrate that GEP can act as a promising biomarker for selecting patients who may benefit from ICB. However, appropriate genetic testing is essential as it could provide comprehensive guide for choosing appropriate ICB treatment. Additional studies are still needed to confirm the exact predictive value of GEP.

## Non-Tumor-Related Biomarkers for ICB

### Tumor Microenvironment-Related Biomarkers

Tumor fate depends on the interplay between tumor and tumor microenvironment. Immune cells, fibroblasts, and cells that comprise the blood vessels constitute the tumor microenvironment that emerges during tumor progression (72). The cancer-immunity cycle remains the theoretical basis behind the advent of tumor immunotherapy. T cell infiltration is one of the key steps in this cycle and plays a vital role in the response of patients to immunotherapy. Several studies have demonstrated that some tumors may display a low level of T cell infiltration into the tumor sites. On the contrary, other tumors may have a large number of T cell infiltration but those T cells can be dysfunctional (23).

CD8^+^ T cells were found to significantly correlate with a better outcome in cancers and could predict the efficacy of ICB (73, 74). Tumeh et al. studied a cohort of samples from metastatic melanoma patients and found that those with a higher CD8^+^ T cell density at the invasive margin before treatment were more likely to respond to pembrolizumab therapy and could also correlate to a radiographic reduction of tumor size. Additional studies also showed that an increased TILs numbers could correlate with response to chemotherapy (75). CD8^+^ TILs can regulate the expression of PD-1/PD-L1 immune inhibitory axis. A higher proportion of PD-1^high^CD8^+^ T cells were found in patients who responded better to anti-PD-1/PD-L1 therapy (76). This observation was consistent with another study that classified tumors into 4 subtypes based on PD-L1 expression and presence of TILs: Type 1 (PD-L1–, TIL–), Type 2(PD-L1+, TIL+), Type 3 (PD-L1–, TIL+), and Type 4(PD-L1+, TIL–), and it was proposed that T2 tumors were more likely to respond to ICB (77). Furthermore, TCR diversity could serve as an optimal predictive parameter to anti-PD1 therapy. A more restricted TCR response could be associated with an accumulation of tumor antigen-specific T cells and a better clinical response to pembrolizumab (73). On the other hand, the Tumor Immune Dysfunction and Exclusion (TIDE) can facilitate tumor immune evasion through integrating the expression signatures of T cell dysfunction and T cell exclusion could accurately predict ICB response in melanoma. The predictive value of TIDE has been reported to be presumably greater than other biomarkers such as PD-L1 expression (23).

While Kaseb et al. showed that CD8^+^ T cell infiltration in HCC can be a predictive biomarker for ICB response (78), another recent study revealed that CD8^+^ T cell infiltration was not enough for predicting the therapeutic efficacy of ICB in HCC (79). Additionally, three different immune subtypes were identified in HCC based on the clinical, molecular and genomic characteristics. Among them, Type A had higher CD8^+^ T cells, but the prognosis was observed to be poor. This was because Type A had more T cell dysfunctionalities, higher expression of immune checkpoints such as PD-1, greater infiltration of immunosuppressive cells, and displayed genomic alterations such as mutations in TP53. All of these factors were found to contribute to poor survival. In support of Liu's finding, another study reported that renal cancer patients had a high infiltration of CD8^+^ T cells, but these patients did not substantially benefit from ICB therapy (80). However, previous studies have primarily focused on the CD8^+^ T cells and ignored the role of CD4^+^ T cells. A recent study has shown that a specific CD4^+^ T cell subset can correlate with the response to ICB in melanoma. This work highlighted the necessity of exploring the relationship between CD4^+^ T cell and anti-tumor immunity (81). Moreover, the status of CD4^+^ T cell in peripheral blood was observed to be associated with PD-1 blockade therapy in NSCLC (82).

Moreover, compared to T cells that essentially serve as a positive prognostic factor in predicting ICB response, other immune cells such as T regulatory cells (Tregs), myeloid-derived suppressor cells (MDSCs), and tumor-associated macrophages (TAMs) can also contribute to the immunosuppressive environment and can be linked to a poor clinical outcome (83). Tregs act mainly by constraining T cell function in part by facilitating the expression of CD8^+^ T cell exhaustion-related gene (84). The development and expansion of Tregs can be regulated by indole 2,3-dioxygenase 1 (IDO1). IDO1 has been shown to suppress T cell infiltration and promote tumor immune evasion (85). MDSCs can exist in two major subtypes: monocytic (M-MDSC) and polymorphonuclear (PMN-MDSC) that can share phenotypic and morphological features with monocytes and neutrophils, respectively. Interestingly, MDSCs not only increase the expression of immunosuppressive molecules, but can also cause T cell exhaustion (86). An accumulation of M-MDSCs in fibrotic livers induced by p38 mitogen activated protein kinase (MAPK) signaling was reported to cause a reduced TILs infiltration and aggressive HCC growth. Inhibition of p38 MAPK could enhance the efficacy of anti-PD1 therapy (87). TAMs are the most abundant immune cells in tumor microenvironment and can be classified into 2 main phenotypes: M1 and M2 TAMs (88, 89). M1 TAMs play a role in killing tumor cells by secreting pro-inflammatory cytokines and expressing nitric oxide synthase (iNOS). Conversely, M2TAMs secrete IL-10, TGF-β, and IL-13 to suppress the immune reaction and can stimulate angiogenesis (90). TAMs can also produce matrix metalloproteinase 9 (MMP9). A higher expression of MMP9 was found to be linked to a better response to ICB therapy but poor overall survival (79).

Tumor immunoscore is a novel method to classify cancers that primarily focuses on T cell infiltration into tumors and could also predict an overall clinical outcome and risk of recurrence in human malignancies (91). The immunoscore can be based on the quantification of CD3^+^ and CD8^+^ T cells both at the tumor center and at the invasive margins. The score, ranges from 0 when low infiltration of both types of the immune cells in both the regions, to 4 when higher densities were found in both of the regions. Immunoscore was initially used to determine the prognosis of stage I/II/ III colorectal cancer patients but was later applied to other cancers such as lung cancer, melanomas and HCCs (92). A recent study emphasized the role of immune cell infiltration rather than PD-L1 status in predicting patient response to ICB therapy (93). This study revealed that patients with a higher immunoscore were more likely to have a higher OS and PFS, but the relationship between immunoscore in metastases and clinical outcome was not observed. Immunoscore could also effectively predict response to chemotherapy in stage III colorectal cancer. Interestingly, patients with a higher immunoscore were more likely to benefit from chemotherapy in terms of recurrent risk (94). Moreover, in rectal cancer, a higher immunoscore was observed to be directly correlated with a greater down-staging after chemoradiotherapy (95).

### Circulating Prognostic Biomarkers

In comparison to other biomarkers, the analysis of circulating markers may be easy, relatively cheap and non-invasive. Immune cell counts, lymphocytes phenotype and serum protein signature such as IL-8 have been suggested to be associated with clinical outcome of cancer patients who were primarily treated with ICB (96–100). In addition, the serum tumor markers and circulating tumor DNA were also observed to be associated with response (101–103).

### Immune Cell Counts and Lymphocytes Phenotype

Many studies have reported that immune cell count can serve as a predictor in melanoma patients treated with ipilimumab (104, 105). Patients with low absolute monocyte and neutrophil count, greater relative-lymphocyte count, high absolute eosinophils count and low neutrophil-to-lymphocyte ratio exhibited a more favorable overall survival and better prognosis (96, 104). Such a relationship between immune cells and clinical outcome also can be found in melanoma patients treated with pembrolizumab (106) or nivolumab and ipilimumab (107). A recent study elucidated the differences between responders and non-responders based on single-cell mass cytometry combined with clustering and regression analyses. It indicated that lower frequencies of CD4^+^T cells, CD8^+^ T cells and γδ T cells but higher frequencies of NK T (NKT) cells and CD19^−^HLA-DR^+^ myeloid cells were present in responder patients. More importantly, the work indicated that the frequency of classical CD14^+^CD16^−^CD33^+^HLA^−^DR^hi^ monocytes might serve as a potential predictive factor in guiding PD-1 blockade immunotherapy (108). Peripheral CD8+ clonality has also shown significant promise as a predictor of response to ICB (109).

The role of NK cells has been poorly explored in immunotherapy. NK cells can show promise as predictive biomarkers in immunotherapy and increased numbers of CD69+ MIP-1β+ NK cells were found in responders compared to non-responders in melanoma (110). Henna Kasanen et al. revealed that NKT cell frequency and NK cell immunophenotype may also possess predictive value in patients treated with anti-PD1 therapy (111). They demonstrated that a high frequency of NKT cells was found in the responders and patients with the highest NKT cells achieved a complete response to anti-PD1 therapy. Additionally, in NSCLC, the NK cell frequency and MDSC subsets could also be used to evaluate the efficacy of PD-1 inhibitors (112).

Peripheral lymphocytes phenotyping has been recently reported as a method to predict clinical responses in cancers treated with ICB. Both the baseline levels of CD45RO^+^/CD8^+^ T cells (113) and CD8 effector-memory type 1 (EM1) T-cells at baseline (114) can serve as potential biomarker candidates for predicting the clinical outcome of ipilimumab treatment in melanoma. The frequency of PD-1+CD56+ T-cells in peripheral blood before immunotherapy may also play an important role in predicting the clinical outcome after ICB therapy in stage IV melanoma (98). A lower frequency of PD-1+CD56+ T-cells was reported to correlate with better clinical response, longer PFS and OS. However, neither the frequencies of CD4+ or CD8+ T-cells without CD56 expression, nor PD-1+ fraction of the CD4 or CD8 subsets were directly found to be associated with clinical benefit. Lower circulating CD8+PD-1+CD73+ lymphocytes were correlated with better outcomes after nivolumab treatment in melanoma (115). In addition, it was reported that CD39+CD8+ T cells can serve as potential predictive and prognostic biomarkers in ICB-treated NSCLC patients (116). Zappasodi et al. found that a subset of CD4+Foxp3– T cells with high PD-1 expression (4PD1hi) can inhibit T cell function and the decreased 4PD1hi frequencies may correlate with favorable clinical outcome after PD-1 blockade in NSCLC (117). The expression levels of LAG-3 and OX40 on peripheral T cells were also found to serve as predictive biomarker for anti-PD-1 therapy in gastric cancer (118).

### Serum Protein Signature

Hardy-Werbin conducted a study to evaluate whether cytokine levels can act as a predictive biomarker of ipilimumab response in small cell lung cancer (SCLC) patients (99). The results showed that SCLC patients may have a lower level of serum effector T helper 1 (Th1), Th2, and pro-inflammatory cytokines as compared with healthy population and the baseline level was associated with age, PS and stage. High levels of IL-2 were found to be related to the sensitivity to ipilimumab, while higher levels of IL-6 and TNFα were associated with resistance to ipilimumab. Additionally, changes in IL-4 levels during immunochemotherapy correlated with a better overall survival. Patients in which IL-4 levels were increased by more than 32% had a better overall survival. Recent studies have also highlighted the predictive value of IL-6 level in NSCLC patients treated with ICB. Kang and colleagues evaluated the relationship between baseline serum level of IL-6 and the possible response to immunotherapy. They demonstrated that the IL-6 level can serve as a potential marker for predicting the clinical benefit of ICI treatment, particularly in patients with no or lower PD-L1 expression. It was observed that patients with lower IL-6 level had a longer median overall survival and a stable disease control rate (119). Moreover, another research measured the change in 12 cytokine levels before and during anti-PD-1 therapy. A decrease in IL-6 level was associated with improved outcome and patients with higher C reactive protein (CRP) levels displayed a shorter PFS. The predictive role of IL-6 and CRP has also been proposed in melanoma (120) and triple-negative breast cancer (121).

Additionally, various ongoing studies have also highlighted the predictive role of IL-8 levels. IL-8 is a member of the CXC chemokine family that can be secreted by malignant cells and tumor stroma cells. The level of IL-8 is correlated with the tumor burden in preclinical models and in patients with cancer (122). Furthermore, the IL-8 levels also correlate with the response to anti-PD-1 treatment in cancer. For instance, Sanmamed et al. demonstrated that the serum IL-8 levels could predict the efficacy of anti-PD-1 therapy in melanoma and NSCLC patients (123). They revealed that early changes in serum IL-8 levels (2–4 weeks after treatment initiation) were significantly associated with beneficial clinical effects of ICB therapy. Patients presenting an early decrease in serum IL-8 levels were found to have longer overall survival than patients with an early increase in levels of this chemokine. IL-8 levels may also be helpful to identify pseudoprogression, and serum IL-8 levels were indeed reported to decrease significantly during pseudoprogression. However, baseline serum IL-8 levels were not associated with the response in this small-scale study. In a larger analysis, elevated baseline serum IL-8 levels correlated with poor outcome in cancer patients treated with ICB therapy (124). This report also identified 23 pgml^−1^ dose as a clinically relevant stratification cut-off. In addition, it was found that the baseline serum IL-8 levels might indicate unfavorable tumor microenvironment conditions. Elevated tumor IL-8 level was also negatively associated with tumor-infiltrating neutrophils and/or monocytes, tumor IFN-γ and T-cell infiltration-related transcript signatures which can contribute to an immunosuppressive environment. However, the level of IL-8 did not directly correlate with PD-L1 expression and TMB. Another larger study also suggested that the serum IL-8 levels could serve as a potential biomarker to stratify patients who may benefit from ICB therapy (125). This finding also revealed that the expression of IL-8 was higher in myeloid clusters than in lymphoid clusters through single-cell RNA sequencing. Moreover, compared with responders, non-responders had a high proportion of IL8-producing myeloid and lymphoid cells, as well as displayed a higher expression of IL-8. Additionally, it was found that the higher IL8 expression correlated with decreased expression of the antigen-presentation machinery which can lead to the reduced response to ICB therapy. Thus, these results underline that the plasma IL-8 may act as a negative prognostic biomarker, it could be considered as an important therapeutic target.

The level of LDH (lactate dehydrogenase) can also serve as a predictor for response to ICB. A study analyzed the outcome of BRAF-mutant metastatic melanoma patients with baseline serum LDH of ≥2× upper limit of normal, who were initially treated with first-line targeted therapy followed by ICB administration. It was found that the median overall survival was significantly higher in patients with normalized LDH at the start of ICB treatment. However, randomized trials are still needed to assess the predictive value (126). The levels of serum acute phase reactants including CRP, serum amyloid A and P, and complement components could also contribute significantly to the treatment stratification in melanoma patients (127). A higher level has been often correlated with poor outcome in patients treated with PD-1 antibodies.

### Serum Tumor Markers

#### Early Alpha-Fetoprotein Response in HCC

AFP, a glycoprotein reported to be expressed by HCC cells, can be found in approximately 70% of HCC patients (128). It not only serves as the most widely used serum biomarker to detect HCC but has also been observed to be associated with response to anti-angiogenic therapy and ICB therapy. A study showed that an early AFP response, with more than 20% reduction in serum AFP level after receiving first 4 weeks of antiangiogenic therapy, could be directly related to the objective response in HCC patients (129). Such an early AFP response (>20% reduction in AFP levels after first 4 weeks of ICB therapy) could accurately predict the efficacy of ICB therapy. For instance, early AFP responders who had pre-treatment AFP level of >20 ng/mL showed significantly longer overall survival and progression-free survival as compared to non-responders (102). In this study, ~50% of AFP non-responders exhibited progressive disease during the first assessment and none of them achieved a complete or partial response later. Interestingly, another study showed that patients with early AFP > 10% reduction were more likely to demonstrate a higher objective response rate and disease control rate (130). However, such a relationship was not found in patients with baseline AFP level <10 ng/ml. It was also observed that better liver reserves (Child–Pugh class A or ALBI grade 1) as well as early AFP response could also serve as good predictors of survival. However, these results were obtained from a small sample size and further validation studies are still needed. Moreover, the levels of serum AFP are normal in 15–30% advanced HCC.

#### Serum Tumor Markers in Lung Cancer

Kang et al. revealed a direct correlation between four tumor markers (CEA, CA125, CYFRA21-1, and SCC-Ag) in advanced NSCLC and the patient response to PD-1/PD-L1 inhibitors (103). The patients in this study were divided into <2/4 biomarkers improvement group and ≥2/4 biomarkers improvement group. At least a 20% decrease from the baseline was considered as a meaningful improvement after 6 weeks of treatment. They found that the patients in ≥2/4 biomarkers improvement group had significantly longer response rate, OS and PFS. Another study has also shown that a reduction in serum levels of CYFRA21-1 or CEA can be associated with favorable clinical outcomes and can be considered as a biomarker to predict the immunotherapy efficacy, but neuron specific enolase (NSE) was not directly related to the efficacy of nivolumab (131).

### Circulating Tumor DNA

Circulating tumor DNA (ctDNA) generally refers to the fraction of cell-free DNA in a patient's blood originating from tumor cells. ctDNA can act as an independent predictor of patient response to ICB. Recent findings have also revealed that ctDNA could also monitor tumor responsiveness to ICB. Lee et al. revealed that melanoma patients with undetected ctDNA levels at baseline or after 8 weeks of therapy had a higher response rate to anti-PD1 therapy and displayed a longer overall survival. On the contrary, an elevated ctDNA upon therapy correlated with a poor prognosis (101). Goldberg and colleagues reported that patients with a drop in ctDNA level are more likely to benefit from ICB and have a superior OS in metastatic NSCLC. Interestingly, a strong correlation has been found between the levels of ctDNA and radiographic response (132), and the efficacy of chemotherapy and target therapies (133). Moreover, in patients with metastatic NSCLC, ctDNA has been approved as a non-invasive alternative to tumor biopsy samples to facilitate the detection of EGFR (epidermal growth factor receptor)-activating mutations (134). However, ctDNA cannot act as an accurate predictor in patients with predominant brain metastases as the blood brain barrier can prevent ctDNA from entering the circulation.

Overall, the level of ctDNA can be considered to have a great potential for predicting the efficacy of ICB. However, further prospective clinical trials are still required to validate the exact relationship between ctDNA analysis and the efficacy of immunotherapy treatment.

### Gut Microbiome

The human gut microbiome is a complicated ecosystem. The composition and function of gut microbiome can be easily influenced by external factors such as stress, lifestyle, and drugs including antibiotics and non-antibiotic. Additionally, recent studies have also highlighted an important role of the gut microbiome in affecting the efficacy of immunotherapy.

Previously, the gut microbiome was reported to be correlated with the efficacy of anti-CTLA-4 therapy in mice, as the efficacy was reduced in germ-free mice or pathogen-free mice administered with antibiotics before anti-CTLA-4 treatment (135). Subsequently, another study revealed that the gut microbiome can influence the efficacy of anti-PD1 therapy (136). Though the underlying mechanisms remain unclear, it is possible that microbial antigens could increase antigen presentation ability and enhance T cell reactivity. Moreover, the gut microbes can infiltrate tumor microenvironment and produce chemotactic factors, which can facilitate the migration of immune cells to tumor sites. These findings have instigated scientists worldwide to explore the relationship between the gut microbiome and clinical outcome of immunotherapy. The significance of dynamic variation of the gut microbiome during immunotherapy has been recently highlighted in case of HCC (137). Using metagenomic sequencing, it was found that fecal samples from anti-PD-1 responders showed a higher taxa richness and greater gene counts than the non-responder. In addition, the beta diversity exhibited a striking difference at week 6. Compared to the composition of the gut microbiome in healthy adults, microbial composition of the responders did not significantly alter during the treatment. However, remarkable changes were noticed in the non-responders. In fact, proteobacteria was increased from week 3, and became predominant at week 12 in these patients. These findings suggested that the composition and dynamic variation of the gut microbiome could also serve as a useful monitoring index to predict patient response to anti-PD1 therapy in HCC patients. It could be inferred that modulating the gut ecosystem may enhance ICB treatment efficacy. Gut microbiota biomarkers may also have an enormous potential to serve as a non-invasive tool for the early diagnosis of HCC (138).

However, there are no uniform standards related to the methods of fecal sample collection, extraction and sequencing of bacterial DNA. As a novel biomarker of ICB response, the gut microbiota must be carefully validated in future prospective studies.

## Conclusion and Future Perspectives

ICB therapy has fundamentally changed the current paradigm of cancer treatment. However, a large number of patients fail to respond to ICB. Immune-related adverse events and high costs can also be considered as potential drawbacks of ICB therapy. Thus, it is imperative to develop robust biomarkers to identify patient populations with a greater likelihood of response to ICB. In this review, several potentially predictive biomarkers, which may help to select patients for ICB, have been discussed. However, all of these biomarkers show certain limitations. Moreover, assessment of all these markers has been challenging, mainly due to a lack of systematic collection of tumor tissue both before, during and after ICB treatment. Proper pre-clinical models have to be developed to validate these distinct biomarkers. Additionally, given the complexity of the immune system, a single marker may not be sufficient to predict an overall response in all patients. These challenges can be overcome by developing more sophisticated strategies. On the other hand, proper identification of accurate biomarkers based on the comprehensive immune profiling of individual tumors needs to be done. Future clinical trials must be designed to incorporate biomarkers analysis to some degree. Biomarkers that allow early assessment of immune activation may prove to be more significant than pretreatment predictive biomarkers. Besides, more accurate detection methods such as multi-omics interrogation and combining different markers will significantly reduce the assumptive risks and provide new avenues.

## Author Contributions

ML put forward the conception, design, and completed critical comments and revision. YL drafted the manuscript. YL, XL, QH, and XZ collected, analyzed data, collected, and prepared table. All authors contributed to the article and approved the submitted version.

## Conflict of Interest

The authors declare that the research was conducted in the absence of any commercial or financial relationships that could be construed as a potential conflict of interest.

## References

[1] Topalian SL Taube JM Pardoll DM. Neoadjuvant checkpoint blockade for cancer immunotherapy. Science. (2020) 367:6477. 10.1126/science.aax0182 32001626 PMC7789854

[2] Chen DS Mellman I. Oncology meets immunology: the cancer-immunity cycle. Immunity. (2013) 39:1–10. 10.1016/j.immuni.2013.07.012 23890059

[3] Qin S Xu L Yi M Yu S Wu K Luo S. Novel immune checkpoint targets: moving beyond PD-1 and CTLA-4. Mol Cancer. (2019) 18:155. 10.1186/s12943-019-1091-2 31690319 PMC6833286

[4] Hodi FS O'Day SJ McDermott DF Weber RW Sosman JA Haanen JB. Improved survival with ipilimumab in patients with metastatic melanoma. N Engl J Med. (2010) 363:711–23. 10.1056/NEJMoa1003466 20525992 PMC3549297

[5] Robert C Ribas A Schachter J Arance A Grob J-J Mortier L. Pembrolizumab versus ipilimumab in advanced melanoma (KEYNOTE-006): post-hoc 5-year results from an open-label, multicentre, randomised, controlled, phase 3 study. Lancet Oncol. (2019) 20:1239–51. 10.1016/S1470-2045(19)30388-2 31345627

[6] Zou W Wolchok JD Chen L. PD-L1 (B7-H1) and PD-1 pathway blockade for cancer therapy: Mechanisms, response biomarkers, and combinations. Sci Transl Med. (2016) 8:328rv324. 10.1126/scitranslmed.aad7118 26936508 PMC4859220

[7] Patel SP Kurzrock R. PD-L1 expression as a predictive biomarker in cancer immunotherapy. Mol Cancer Therap. (2015) 14:847–56. 10.1158/1535-7163.MCT-14-0983 25695955

[8] Daud AI Wolchok JD Robert C Hwu W-J Weber JS. Programmed death-ligand 1 expression and response to the anti-programmed death 1 antibody pembrolizumab in melanoma. J Clin Oncol.(2016) 34:4102–9. 10.1200/JCO.2016.67.2477 27863197 PMC5562434

[9] Cha JH Chan LC Li CW Hsu JL Hung MC. Mechanisms controlling PD-L1 expression in cancer. Mol Cell. (2019) 76:359–70. 10.1016/j.molcel.2019.09.030 31668929 PMC6981282

[10] Herbst RS Soria J-C Kowanetz M Fine GD Hamid O Gordon MS. Predictive correlates of response to the anti-PD-L1 antibody MPDL3280A in cancer patients. Nature. (2014) 515:563–7. 10.1038/nature14011 25428504 PMC4836193

[11] Taube JM Klein A Brahmer JR Xu H Pan X Kim JH. Association of PD-1, PD-1 ligands, and other features of the tumor immune microenvironment with response to anti-PD-1 therapy. Clin Cancer Res. (2014) 20:5064–74. 10.1158/1078-0432.CCR-13-3271 24714771 PMC4185001

[12] Garon EB Rizvi NA Hui R Leighl N Balmanoukian AS Eder JP. Pembrolizumab for the treatment of non-small-cell lung cancer. N Engl J Med. (2015) 372:2018–28. 10.1056/NEJMoa1501824 25891174

[13] Schmid P Adams S Rugo HS Schneeweiss A Barrios CH Iwata H. Atezolizumab and nab-paclitaxel in advanced triple-negative breast cancer. N Engl J Med. (2018) 379:2108–21. 10.1056/NEJMoa1809615 30345906

[14] El-Khoueiry AB Sangro B Yau T Crocenzi TS Kudo M Hsu C. Nivolumab in patients with advanced hepatocellular carcinoma (CheckMate 040): an open-label, non-comparative, phase 1/2 dose escalation and expansion trial. Lancet. (2017) 389:2492–502. 10.1016/S0140-6736(17)31046-2 28434648 PMC7539326

[15] Sharma P Callahan MK Bono P Kim J Spiliopoulou P Calvo E. Nivolumab monotherapy in recurrent metastatic urothelial carcinoma (CheckMate 032): a multicentre, open-label, two-stage, multi-arm, phase 1/2 trial. Lancet Oncol. (2016) 17:1590–8. 10.1016/S1470-2045(16)30496-X 27733243 PMC5648054

[16] Davis AA Patel VG. The role of PD-L1 expression as a predictive biomarker: an analysis of all US Food and Drug Administration (FDA) approvals of immune checkpoint inhibitors. J Immunother Cancer. (2019) 7:278. 10.1186/s40425-019-0768-9 31655605 PMC6815032

[17] Meng X Huang Z Teng F Xing L Yu J. Predictive biomarkers in PD-1/PD-L1 checkpoint blockade immunotherapy. Cancer Treat Rev. (2015) 41:868–76. 10.1016/j.ctrv.2015.11.001 26589760

[18] Powles T Eder JP Fine GD Braiteh FS Loriot Y Cruz C. MPDL3280A (anti-PD-L1) treatment leads to clinical activity in metastatic bladder cancer. Nature. (2014) 515:558–62. 10.1038/nature13904 25428503

[19] Kleinovink JW Marijt KA Schoonderwoerd MJA van Hall T Ossendorp F Fransen MF. PD-L1 expression on malignant cells is no prerequisite for checkpoint therapy. Oncoimmunology. (2017) 6:e1294299. 10.1080/2162402X.2017.1294299 28507803 PMC5414865

[20] Shen YC Hsu CL Jeng YM Ho MC Ho CM Yeh CP. Reliability of a single-region sample to evaluate tumor immune microenvironment in hepatocellular carcinoma. J Hepatol. (2020) 72:489–97. 10.1016/j.jhep.2019.09.032 31634533

[21] Bodor JN Boumber Y Borghaei H. Biomarkers for immune checkpoint inhibition in non-small cell lung cancer (NSCLC). Cancer. (2020) 126:260–70. 10.1002/cncr.32468 31691957 PMC7372560

[22] Itoh S Yoshizumi T Yugawa K Imai D Yoshiya S Takeishi K. Impact of immune response on outcomes in hepatocellular carcinoma: association with vascular formation. Hepatol. (2020) 72:1987–99. 10.1002/hep.31206 32112577

[23] Jiang P Gu S Pan D Fu J Sahu A Hu X. Signatures of T cell dysfunction and exclusion predict cancer immunotherapy response. Nat Med. (2018) 24:1550–8. 10.1038/s41591-018-0136-1 30127393 PMC6487502

[24] Liu D Schilling B Liu D Sucker A Livingstone E Jerby-Arnon L. Integrative molecular and clinical modeling of clinical outcomes to PD1 blockade in patients with metastatic melanoma. Nat Med. (2019) 25:1916–27. 10.1038/s41591-019-0654-5 31792460 PMC6898788

[25] Yarchoan M Albacker LA Hopkins AC Montesion M Murugesan K Vithayathil TT. PD-L1 expression and tumor mutational burden are independent biomarkers in most cancers. JCI insight. (2019) 4:e126908. 10.1172/jci.insight.126908 30895946 PMC6482991

[26] Chan TA Yarchoan M Jaffee E Swanton C Quezada SA Stenzinger A. Development of tumor mutation burden as an immunotherapy biomarker: utility for the oncology clinic. Ann Oncol. (2019) 30:44–56. 10.1093/annonc/mdy495 30395155 PMC6336005

[27] Snyder A Makarov V Merghoub T Yuan J Zaretsky JM Desrichard A. Genetic basis for clinical response to CTLA-4 blockade in melanoma. N Engl J Med. (2014) 371:2189–99. 10.1056/NEJMoa1406498 25409260 PMC4315319

[28] Fumet J-D Truntzer C Yarchoan M Ghiringhelli F. Tumour mutational burden as a biomarker for immunotherapy: current data and emerging concepts. Europ J Cancer. (2020) 131:40–50. 10.1016/j.ejca.2020.02.038 32278982 PMC9473693

[29] Rizvi NA Hellmann MD Snyder A Kvistborg P Makarov V Havel JJ. Cancer immunology. Mutational landscape determines sensitivity to PD-1 blockade in non-small cell lung cancer. Science. (2015) 348:124–8. 10.1126/science.aaa1348 25765070 PMC4993154

[30] Hellmann MD Nathanson T Rizvi H Creelan BC Sanchez-Vega F Ahuja A. Genomic features of response to combination immunotherapy in patients with advanced non-small-cell lung Cancer. Cancer Cell. (2018) 33:843–52 e844. 10.1016/j.ccell.2018.03.018 29657128 PMC5953836

[31] Hellmann MD Callahan MK Awad MM Calvo E Ascierto PA Atmaca A. Tumor mutational burden and efficacy of nivolumab monotherapy and in combination with ipilimumab in small-cell lung cancer. Cancer Cell. (2018) 33:853–61 e854. 10.1016/j.ccell.2018.04.001 29731394 PMC6750707

[32] Rosenberg JE Hoffman-Censits J Powles T van der Heijden MS Balar AV Necchi A. Atezolizumab in patients with locally advanced and metastatic urothelial carcinoma who have progressed following treatment with platinum-based chemotherapy: a single-arm, multicentre, phase 2 trial. Lancet (London, England). (2016) 387:1909–20. 10.1016/S0140-6736(16)00561-4 26952546 PMC5480242

[33] Powles T Durán I van der Heijden MS Loriot Y Vogelzang NJ De Giorgi U. Atezolizumab versus chemotherapy in patients with platinum-treated locally advanced or metastatic urothelial carcinoma (IMvigor211): a multicentre, open-label, phase 3 randomised controlled trial. Lancet (London, England). (2018) 391:748–57. 10.1016/S0140-6736(17)33297-X 29268948

[34] Wolf Y Bartok O Patkar S Eli G Cohen S Litchfield K. UVB-induced tumor heterogeneity diminishes immune response in melanoma. Cell. (2019) 179:219–35.e221. 10.1016/j.cell.2019.08.032 31522890 PMC6863386

[35] McGranahan N Furness AJ Rosenthal R Ramskov S Lyngaa R Saini SK. Clonal neoantigens elicit T cell immunoreactivity and sensitivity to immune checkpoint blockade. Science. (2016) 351:1463–9. 10.1126/science.aaf1490 26940869 PMC4984254

[36] Yarchoan M Hopkins A Jaffee EM. Tumor mutational burden and response rate to PD-1 inhibition. N Engl J Med. (2017) 377:2500–1. 10.1056/NEJMc1713444 29262275 PMC6549688

[37] Gunjur A. Nivolumab plus ipilimumab in advanced renal-cell carcinoma. Lancet Oncol. (2018) 19:e232. 10.1016/S1470-2045(18)30257-2 29606588

[38] Knepper TC Montesion M Russell JS Sokol ES Frampton GM Miller VA. The genomic landscape of merkel cell carcinoma and clinicogenomic biomarkers of response to immune checkpoint inhibitor therapy. Clin Cancer Res. (2019) 25:5961–71. 10.1158/1078-0432.CCR-18-4159 31399473 PMC6774882

[39] Langer C Gadgeel S Borghaei H Patnaik A Papadimitrakopoulou V. OA04.05 KEYNOTE-021: TMB outcomes for carboplatin pemetrexed with or without pembrolizumab for nonsquamous NSCLC. J Thora Oncol. (2019) 14:S216. 10.1016/j.jtho.2019.08.426

[40] Fancello L Gandini S Pelicci PG Mazzarella L. Tumor mutational burden quantification from targeted gene panels: major advancements and challenges. J Immunother Cancer. (2019) 7:183. 10.1186/s40425-019-0647-4 31307554 PMC6631597

[41] American Association for Cancer Research (AACR). TMB faces validation hurdles. Cancer Disc. (2019) 9:1334. 10.1158/2159-8290.Cd-nd2019-010 31462395

[42] Le DT Uram JN Wang H Bartlett BR Kemberling H Eyring AD. PD-1 blockade in tumors with mismatch-repair deficiency. N Engl J Med. (2015) 372:2509–20. 10.1056/NEJMoa1500596 26028255 PMC4481136

[43] Marabelle A Le DT Ascierto PA Di Giacomo AM De Jesus-Acosta A Delord P. Efficacy of pembrolizumab in patients with noncolorectal high microsatellite instability/mismatch repair-deficient cancer: results from the phase II KEYNOTE-158 study. J Clin Oncol. (2020) 38:1–10. 10.1200/JCO.19.02105 31682550 PMC8184060

[44] Boyiadzis MM Kirkwood JM Marshall JL Pritchard CC Azad NS Gulley JL. Significance and implications of FDA approval of pembrolizumab for biomarker-defined disease. J Immunother Cancer. (2018) 6:35. 10.1186/s40425-018-0342-x 29754585 PMC5950135

[45] Lemery S Keegan P Pazdur R. First FDA approval agnostic of cancer site - when a biomarker defines the indication. N Engl J Med. (2017) 377:1409–12. 10.1056/NEJMp1709968 29020592

[46] Overman MJ McDermott R Leach JL Lonardi S Lenz H-J Morse MA. Nivolumab in patients with metastatic DNA mismatch repair-deficient or microsatellite instability-high colorectal cancer (CheckMate 142): an open-label, multicentre, phase 2 study. Lancet Oncol. (2017) 18:1182–91. 10.1016/S1470-2045(17)30422-9 28734759 PMC6207072

[47] Marabelle A Le D Ascierto P Di Giacomo A De Jesus-Acosta A Delord J. Efficacy of pembrolizumab in patients with noncolorectal high microsatellite instability/mismatch repair-deficient cancer: results from the phase II KEYNOTE-158 study. J Clin Oncol. (2020) 38:1–10. 10.1200/jco.19.02105 31682550 PMC8184060

[48] Azad N Gray R Overman M Schoenfeld J Mitchell E Zwiebel J. Nivolumab is effective in mismatch repair-deficient noncolorectal cancers: results from Arm Z1D-A subprotocol of the NCI-MATCH (EAY131) study. J Clin Oncol. (2020) 38:214–22. 10.1200/jco.19.00818 31765263 PMC6968795

[49] Mandal R Samstein RM Lee KW Havel JJ Wang H Krishna C. Genetic diversity of tumors with mismatch repair deficiency influences anti-PD-1 immunotherapy response. Science. (2019) 364:485–91. 10.1126/science.aau0447 31048490 PMC6685207

[50] Abida W Cheng ML Armenia J Middha S Autio KA Vargas HA. Analysis of the prevalence of microsatellite instability in prostate cancer and response to immune checkpoint blockade. JAMA Oncol. (2019) 5:471–8. 10.1001/jamaoncol.2018.5801 30589920 PMC6459218

[51] Arora A Olshen AB Seshan VE Shen R. Pan-cancer identification of clinically relevant genomic subtypes using outcome-weighted integrative clustering. Genome Med. (2020) 12:110. 10.1186/s13073-020-00804-8 33272320 PMC7716509

[52] Yi M Jiao D Xu H Liu Q Zhao W Han X. Biomarkers for predicting efficacy of PD-1/PD-L1 inhibitors. Mol Cancer. (2018) 17:129. 10.1186/s12943-018-0864-3 30139382 PMC6107958

[53] Abril-Rodriguez G Ribas A. SnapShot: immune checkpoint inhibitors. Cancer cell. (2017) 31:848–8.e1. 10.1016/j.ccell.2017.05.010 28609660

[54] Ayers M Lunceford J Nebozhyn M Murphy E Loboda A Kaufman DR. IFN-γ-related mRNA profile predicts clinical response to PD-1 blockade. J Clin Invest. (2017) 127:2930–40. 10.1172/JCI91190 28650338 PMC5531419

[55] Fehrenbacher L Spira A Ballinger M Kowanetz M Vansteenkiste J Mazieres J. Atezolizumab versus docetaxel for patients with previously treated non-small-cell lung cancer (POPLAR): a multicentre, open-label, phase 2 randomised controlled trial. Lancet (London, England). (2016) 387:1837–46. 10.1016/S0140-6736(16)00587-0 26970723

[56] Fuchs CS Doi T Jang RW Muro K Satoh T Machado M. Safety and efficacy of pembrolizumab monotherapy in patients with previously treated advanced gastric and gastroesophageal junction cancer: phase 2 Clinical KEYNOTE-059 trial. JAMA Oncol. (2018) 4:e180013. 10.1001/jamaoncol.2018.0013 29543932 PMC5885175

[57] Gainor JF Shaw AT Sequist LV Fu X Azzoli CG Piotrowska Z. EGFR mutations and ALK rearrangements are associated with low response rates to pd-1 pathway blockade in non-small cell lung cancer: a retrospective analysis. Clin Cancer Res. (2016) 22:4585–93. 10.1158/1078-0432.CCR-15-3101 27225694 PMC5026567

[58] Dong Z-Y Zhong W-Z Zhang X-C Su J Xie Z Liu S-Y. Potential predictive value of and mutation status for response to PD-1 blockade immunotherapy in lung adenocarcinoma. Clin Cancer Res. (2017) 23:3012–24. 10.1158/1078-0432.CCR-16-2554 28039262

[59] Skoulidis F Byers LA Diao L Papadimitrakopoulou VA Tong P Izzo J. Co-occurring genomic alterations define major subsets of KRAS-mutant lung adenocarcinoma with distinct biology, immune profiles, therapeutic vulnerabilities. Cancer Disc. (2015) 5:860–77. 10.1158/2159-8290.CD-14-1236 26069186 PMC4527963

[60] Skoulidis F Goldberg ME Greenawalt DM Hellmann MD Awad MM Gainor JF. Mutations and PD-1 inhibitor resistance in -mutant lung adenocarcinoma. Cancer Disc. (2018) 8:822–35. 10.1158/2159-8290.CD-18-0099 PMC6030433 29773717

[61] Thommen DS Koelzer VH Herzig P Roller A Trefny M Dimeloe S. A transcriptionally and functionally distinct PD-1 CD8 T cell pool with predictive potential in non-small-cell lung cancer treated with PD-1 blockade. Nat Med. (2018) 24:994–1004. 10.1038/s41591-018-0057-z 29892065 PMC6110381

[62] Liu X-D Kong W Peterson CB McGrail DJ Hoang A Zhang X. PBRM1 loss defines a nonimmunogenic tumor phenotype associated with checkpoint inhibitor resistance in renal carcinoma. Nat Commun. (2020) 11:2135. 10.1038/s41467-020-15959-6 32358509 PMC7195420

[63] Sade-Feldman M Jiao YJ Chen JH Rooney MS Barzily-Rokni M Eliane J-P. Resistance to checkpoint blockade therapy through inactivation of antigen presentation. Nat Commun. (2017) 8:1136. 10.1038/s41467-017-01062-w 29070816 PMC5656607

[64] Ruiz de Galarreta M Bresnahan E Molina-Sánchez P Lindblad KE Maier B Sia D. β-catenin activation promotes immune escape and resistance to anti-PD-1 therapy in hepatocellular carcinoma. Cancer Disc. (2019) (2019) 9:1124–41. 10.1158/2159-8290.CD-19-0074 31186238 PMC6677618

[65] Nishida N Kudo M. Immune phenotype and immune checkpoint inhibitors for the treatment of human hepatocellular carcinoma. Cancers. (2020) 12:1274. 10.3390/cancers12051274 32443599 PMC7281618

[66] Harding JJ Nandakumar S Armenia J Khalil DN Albano M Ly M. Prospective genotyping of hepatocellular carcinoma: clinical implications of next-generation sequencing for matching patients to targeted and immune therapies. Clin Cancer Res. (2019) 25:2116–26. 10.1158/1078-0432.CCR-18-2293 30373752 PMC6689131

[67] Mo Z Wang Y Cao Z Li P Zhang S. An integrative analysis reveals the underlying association between CTNNB1 mutation and immunotherapy in hepatocellular carcinoma. Front Oncol. (2020) 10:853. 10.3389/fonc.2020.00853 32596147 PMC7304048

[68] Wang L Yan K Zhou J Zhang N Wang M Song J. Relationship of liver cancer with LRP1B or TP53 mutation and tumor mutation burden and survival. J Clin Oncol. (2019) 37(15_suppl):1573–3. 10.1200/JCO.2019.37.15_suppl.1573

[69] Ou Q Yu Y Li A Chen J Yu T Xu X. Association of survival and genomic mutation signature with immunotherapy in patients with hepatocellular carcinoma. Ann Transl Med. (2020) 8:230. 10.21037/atm.2020.01.32 32309377 PMC7154492

[70] Sade-Feldman M Yizhak K Bjorgaard SL Ray JP de Boer CG Jenkins RW. Defining T cell states associated with response to checkpoint immunotherapy in melanoma. Cell. (2018) 175:998–1013.e20. 10.1016/j.cell.2018.10.038 30388456 PMC6641984

[71] Jerby-Arnon L Shah P Cuoco MS Rodman C Su M-J. A cancer cell program promotes T cell exclusion and resistance to checkpoint blockade. Cell. (2018) 75:175. 10.1016/j.cell.2018.09.006 30388455 PMC6410377

[72] Yang L Li A Lei Q Zhang Y. Tumor-intrinsic signaling pathways: key roles in the regulation of the immunosuppressive tumor microenvironment. J Hemat Oncol. (2019) 12:125. 10.1186/s13045-019-0804-8 31775797 PMC6880373

[73] Tumeh PC Harview CL Yearley JH Shintaku IP Taylor EJ Robert L. PD-1 blockade induces responses by inhibiting adaptive immune resistance. Nature. (2014) 515:568–71. 10.1038/nature13954 25428505 PMC4246418

[74] Huang AC Postow MA Orlowski RJ Mick R Bengsch B Manne S. T-cell invigoration to tumour burden ratio associated with anti-PD-1 response. Nature. (2017) 545:60–5. 10.1038/nature22079 28397821 PMC5554367

[75] Danilova L Wang H Sunshine J Kaunitz GJ Cottrell TR Xu H. Association of PD-1/PD-L axis expression with cytolytic activity, mutational load, prognosis in melanoma other solid tumors. Proc Natl Acad Sci USA. (2016) 113:E7769–77. 10.1073/pnas.1607836113 27837027 PMC5137776

[76] Macek Jilkova Z Aspord C Kurma K Granon A Sengel C Sturm N. Immunologic features of patients with advanced hepatocellular carcinoma before and during sorafenib or anti-programmed death-1/programmed death-l1 treatment. Clin Transl Gastroent. (2019) 10:e00058. 10.14309/ctg.0000000000000058 31295151 PMC6708670

[77] Zhang Y Chen L. Classification of advanced human cancers based on tumor immunity in the microenvironment (TIME) for cancer immunotherapy. JAMA Oncol. (2016) 2:1403–4. 10.1001/jamaoncol.2016.2450 27490017 PMC5492376

[78] Kaseb AO Vence L Blando J Yadav SS Ikoma N Pestana RC. Immunologic correlates of pathologic complete response to preoperative immunotherapy in hepatocellular carcinoma. Cancer Immunol Res. (2019) 7:1390–5. 10.1158/2326-6066.CIR-18-0605 31289040 PMC7726707

[79] Liu F Qin L Liao Z Song J Yuan C Liu Y. Microenvironment characterization and multi-omics signatures related to prognosis and immunotherapy response of hepatocellular carcinoma. Exp Hematol Oncol. (2020) 9:10. 10.1186/s40164-020-00165-3 32509418 PMC7249423

[80] Remark R Alifano M Cremer I Lupo A Dieu-Nosjean M-C Riquet M. Characteristics and clinical impacts of the immune environments in colorectal and renal cell carcinoma lung metastases: influence of tumor origin. Clin Cancer Res. (2013) 19:4079–91. 10.1158/1078-0432.CCR-12-3847 23785047

[81] Spitzer MH Carmi Y Reticker-Flynn NE Kwek SS Madhireddy D Martins MM. Systemic immunity is required for effective cancer immunotherapy. Cell. (2017) 168:e15. 10.1016/j.cell.2016.12.022 28111070 PMC5312823

[82] Kagamu H Kitano S Yamaguchi O Yoshimura K Horimoto K Kitazawa M. CD4 T-cell immunity in the peripheral blood correlates with response to anti-pd-1 therapy. Cancer Immunol Res. (2020) 8:334–44. 10.1158/2326-6066.CIR-19-0574 31871122

[83] Sachdeva M Arora SK. Prognostic role of immune cells in hepatocellular carcinoma. EXCLI J. (2020) 19:718–33. 10.17179/excli2020-1455 32636725 PMC7332804

[84] Jiang Y Lo AWI Wong A Chen W Wang Y Lin L. Prognostic significance of tumor-infiltrating immune cells and PD-L1 expression in esophageal squamous cell carcinoma. Oncotarget. (2017) 8:30175–89. 10.18632/oncotarget.15621 28404915 PMC5444735

[85] Zhu H Gu Y Xue Y Yuan M Cao X Liu Q. CXCR2(+) MDSCs promote breast cancer progression by inducing EMT and activated T cell exhaustion. Oncotarget. (2017) 8:114554–67. 10.18632/oncotarget.23020 29383101 PMC5777713

[86] Sawant DV Yano H Chikina M Zhang Q Liao M Liu C. Adaptive plasticity of IL-10(+) and IL-35(+) Treg cells cooperatively promotes tumor T cell exhaustion. Nat Immunol. (2019) 20:724–35. 10.1038/s41590-019-0346-9 30936494 PMC6531353

[87] Liu M Zhou J Liu X Feng Y Yang W Wu F. Targeting monocyte-intrinsic enhancer reprogramming improves immunotherapy efficacy in hepatocellular carcinoma. Gut. (2020) 69:365–79. 10.1136/gutjnl-2018-317257 31076403

[88] Deng R Lu J Liu X Peng X Wang J Li X. PD-L1 expression is highly associated with tumor-associated macrophage infiltration in nasopharyngeal carcinoma. Cancer Manag Res. (2020) 12:11585–96. 10.2147/cmar.S274913 33209062 PMC7669506

[89] Licarete E Rauca V Luput L Drotar D Stejerean I Patras L. Overcoming intrinsic doxorubicin resistance in melanoma by anti-angiogenic and anti-metastatic effects of liposomal prednisolone phosphate on tumor microenvironment. Int J Mol Sci. (2020) 21:2968. 10.3390/ijms21082968 32340166 PMC7215436

[90] Oya Y Hayakawa Y Koike K. Tumor microenvironment in gastric cancers. Cancer Sci. (2020) 111:2696–707. 10.1111/cas.14521 32519436 PMC7419059

[91] Angell H Galon J. From the immune contexture to the Immunoscore: the role of prognostic and predictive immune markers in cancer. Curr Opin Immunol. (2013) 25:261–7. 10.1016/j.coi.2013.03.004 23579076

[92] Ros-Martínez S Navas-Carrillo D Alonso-Romero JL Orenes-Piñero E. Immunoscore: a novel prognostic tool. Association with clinical outcome, response to treatment and survival in several malignancies. Crit Rev Clin Lab Sci. (2020) 57:432–43. 10.1080/10408363.2020.1729692 32175789

[93] Kümpers C Jokic M Haase O Offermann A Vogel W Grätz V. Immune cell infiltration of the primary tumor, not PD-L1 status, is associated with improved response to checkpoint inhibition in metastatic Melanoma. Front Med (Lausanne). (2019) 6:27. 10.3389/fmed.2019.00027 30931305 PMC6425878

[94] Mlecnik B Bifulco C Bindea G Marliot F Lugli A Lee JJ. multicenter international society for immunotherapy of cancer study of the consensus immunoscore for the prediction of survival and response to chemotherapy in stage iii colon cancer. J Clin Oncol. (2020) 38:3638–51. 10.1200/JCO.19.03205 32897827 PMC7605397

[95] Anitei M-G Zeitoun G Mlecnik B Marliot F Haicheur N Todosi A-M. Prognostic and predictive values of the immunoscore in patients with rectal cancer. Clin Cancer Res. (2014) 20:1891–9. 10.1158/1078-0432.CCR-13-2830 24691640

[96] Ferrucci PF Ascierto PA Pigozzo J Del Vecchio M Maio M Antonini Cappellini GC. Baseline neutrophils and derived neutrophil-to-lymphocyte ratio: prognostic relevance in metastatic melanoma patients receiving ipilimumab. Ann Oncol. (2016) 27:732–8. 10.1093/annonc/mdw016 26802161

[97] Martens A Wistuba-Hamprecht K Yuan J Postow MA Wong P Capone M. Increases in absolute lymphocytes and circulating CD4+ and CD8+ T cells are associated with positive clinical outcome of melanoma patients treated with ipilimumab. Clin Cancer Res. (2016) 22:4848–58. 10.1158/1078-0432.CCR-16-0249 27169993 PMC5544386

[98] Bochem J Zelba H Amaral T Spreuer J Soffel D Eigentler T. Peripheral PD-1+CD56+ T-cell frequencies correlate with outcome in stage IV melanoma under PD-1 blockade. PLoS ONE. (2019) 14:e0221301. 10.1371/journal.pone.0221301 31419253 PMC6697319

[99] Hardy-Werbin M Rocha P Arpi O Taus Á Nonell L Durán X. Serum cytokine levels as predictive biomarkers of benefit from ipilimumab in small cell lung cancer. Oncoimmunology. (2019) 8:e1593810. 10.1080/2162402X.2019.1593810 31069160 PMC6492977

[100] Teijeira A Garasa S Ochoa MC Villalba Esparza M Olivera I Cirella A. Interleukin-8, Neutrophils, and NETs in a Collusion against Cancer Immunity and Immunotherapy. Clin Cancer Res. (2020) clincanres. 1319. 2020. 10.1158/1078-0432.CCR-20-1319 33376096

[101] Lee JH Long GV Boyd S Lo S Menzies AM Tembe V. Circulating tumour DNA predicts response to anti-PD1 antibodies in metastatic melanoma. Ann Oncol. (2017) 28:1130–6. 10.1093/annonc/mdx026 28327969

[102] Shao Y-Y Liu T-H Hsu C Lu L-C Shen Y-C Lin Z-Z. Early alpha-foetoprotein response associated with treatment efficacy of immune checkpoint inhibitors for advanced hepatocellular carcinoma. Liver Int. (2019) 39:2184–9. 10.1111/liv.14210 31400295

[103] Zhang Z Yuan F Chen R Li Y Ma J Yan X. Dynamics of serum tumor markers can serve as a prognostic biomarker for chinese advanced non-small cell lung cancer patients treated with immune checkpoint inhibitors. Front Immunol. (2020) 11:1173. 10.3389/fimmu.2020.01173 32587591 PMC7298878

[104] Martens A Wistuba-Hamprecht K Geukes Foppen M Yuan J Postow MA Wong P. Baseline peripheral blood biomarkers associated with clinical outcome of advanced melanoma patients treated with ipilimumab. Clin Cancer Res. (2016) 22:2908–18. 10.1158/1078-0432.CCR-15-2412 26787752 PMC5770142

[105] Postow MA Chasalow SD Kuk D Panageas KS Cheng ML Yuan J. Absolute lymphocyte count as a prognostic biomarker for overall survival in patients with advanced melanoma treated with ipilimumab. Melanoma Res. (2020) 30:71–5. 10.1097/CMR.0000000000000633 31425479 PMC7485135

[106] Weide B Martens A Hassel JC Berking C Postow MA Bisschop K. Baseline biomarkers for outcome of melanoma patients treated with pembrolizumab. Clin Cancer Res. (2016) 22:5487–96. 10.1158/1078-0432.CCR-16-0127 27185375 PMC5572569

[107] Rosner S Kwong E Shoushtari AN Friedman CF Betof AS Brady MS. Peripheral blood clinical laboratory variables associated with outcomes following combination nivolumab and ipilimumab immunotherapy in melanoma. Cancer medicine. (2018) 7:690–7. 10.1002/cam4.1356 29468834 PMC5852343

[108] Krieg C Nowicka M Guglietta S Schindler S Hartmann FJ Weber LM. High-dimensional single-cell analysis predicts response to anti-PD-1 immunotherapy. Nat Med. (2018) 24:144–53. 10.1038/nm.4466 29309059

[109] Fairfax BP Taylor CA Watson RA Nassiri I Danielli S Fang H. Peripheral CD8 T cell characteristics associated with durable responses to immune checkpoint blockade in patients with metastatic melanoma. Nat Med. (2020) 26:193–9. 10.1038/s41591-019-0734-6 32042196 PMC7611047

[110] Subrahmanyam PB Dong Z Gusenleitner D Giobbie-Hurder A Severgnini M Zhou J. Distinct predictive biomarker candidates for response to anti-CTLA-4 and anti-PD-1 immunotherapy in melanoma patients. J Immunothe Cancer. (2018) 6:18. 10.1186/s40425-018-0328-8 29510697 PMC5840795

[111] Kasanen H Hernberg M Mäkel,ä S Brück O Juteau S Kohtamäki L. Age-associated changes in the immune system may influence the response to anti-PD1 therapy in metastatic melanoma patients. Cancer Immunol Immunother. (2020) 69:717–30. 10.1007/s00262-020-02497-9 32036449 PMC7183505

[112] Youn J-I Park S-M Park S Kim G Lee H-J Son J. Peripheral natural killer cells and myeloid-derived suppressor cells correlate with anti-PD-1 responses in non-small cell lung cancer. Scient Rep. (2020) 10:9050. 10.1038/s41598-020-65666-x 32493990 PMC7270107

[113] Tietze JK Angelova D Heppt MV Reinholz M Murphy WJ Spannagl M. The proportion of circulating CD45ROCD8 memory T cells is correlated with clinical response in melanoma patients treated with ipilimumab. Europ J Cancer. (2017) 75:268–79. 10.1016/j.ejca.2016.12.031 28242504

[114] Wistuba-Hamprecht K Martens A Heubach F Romano E Geukes Foppen M Yuan J. Peripheral CD8 effector-memory type 1 T-cells correlate with outcome in ipilimumab-treated stage IV melanoma patients. Europ J Cancer. (2017) 73:61–70. 10.1016/j.ejca.2016.12.011 28167454 PMC5599126

[115] Capone M Fratangelo F Giannarelli D Sorrentino C Turiello R Zanotta S. Frequency of circulating CD8+CD73+T cells is associated with survival in nivolumab-treated melanoma patients. J Transl Med. (2020) 18:121. 10.1186/s12967-020-02285-0 32160899 PMC7065327

[116] Koh J Kim Y Lee KY Hur JY Kim MS Kim B. MDSC subtypes and CD39 expression on CD8 T cells predict the efficacy of anti-PD-1 immunotherapy in patients with advanced NSCLC. Europ J Immunol. (2020) 50:1810–9. 10.1002/eji.202048534 32510574 PMC7689686

[117] Zappasodi R Budhu S Hellmann MD Postow MA Senbabaoglu Y Manne S. Non-conventional inhibitory CD4Foxp3PD-1 T cells as a biomarker of immune checkpoint blockade activity. Cancer Cell. (2018) 34:691. 10.1016/j.ccell.2018.09.007 30300585 PMC6656529

[118] Ohmura H Yamaguchi K Hanamura F Ito M Makiyama A Uchino K. OX40 and LAG3 are associated with better prognosis in advanced gastric cancer patients treated with anti-programmed death-1 antibody. Br J Cancer. (2020) 122:1507–17. 10.1038/s41416-020-0810-1 32203221 PMC7217874

[119] Kang DH Park C-K Chung C Oh I-J Kim Y-C Park D. Baseline serum interleukin-6 levels predict the response of patients with advanced non-small cell lung cancer to PD-1/PD-L1 inhibitors. Immune Netw. (2020) 20:e27. 10.4110/in.2020.20.e27 32655975 PMC7327149

[120] Weber JS Tang H Hippeli L Qian M Hodi FS. Serum IL-6 and CRP as prognostic factors in melanoma patients receiving single agent and combination checkpoint inhibition. J Clin Oncol. (2019) 37:100. 10.1200/JCO.2019.37.15_suppl.100

[121] Li Y Fass,ò M Emens LA Molinero L. Abstract CT001: Biomarkers of systemic inflammation associated to reduced clinical activity of atezolizumab monotherapy in patients with metastatic triple negative breast cancer. In Proceedings: AACR Annual Meeting 2019 March 29-April 3, Atlanta, GA (2019).

[122] Sanmamed MF Carranza-Rua O Alfaro C Oñate C Martín-Algarra S Perez G. Serum interleukin-8 reflects tumor burden and treatment response across malignancies of multiple tissue origins. Clin Cancer Res. (2014) 20:5697–707. 10.1158/1078-0432.CCR-13-3203 25224278

[123] Sanmamed MF Perez-Gracia JL Schalper KA Fusco JP Gonzalez A Rodriguez-Ruiz ME. Changes in serum interleukin-8 (IL-8) levels reflect and predict response to anti-PD-1 treatment in melanoma and non-small-cell lung cancer patients. Ann Oncol. (2017) 28:1988–95. 10.1093/annonc/mdx190 28595336 PMC5834104

[124] Schalper KA Carleton M Zhou M Chen T Feng Y Huang S-P. Elevated serum interleukin-8 is associated with enhanced intratumor neutrophils and reduced clinical benefit of immune-checkpoint inhibitors. Nat Med. (2020) 26:688–92. 10.1038/s41591-020-0856-x 32405062 PMC8127102

[125] Yuen KC Liu L-F Gupta V Madireddi S Keerthivasan S Li C. High systemic and tumor-associated IL-8 correlates with reduced clinical benefit of PD-L1 blockade. Nat Med. (2020) 26:693–8. 10.1038/s41591-020-0860-1 32405063 PMC8286544

[126] Schouwenburg MG Suijkerbuijk KPM Koornstra RHT Jochems A van Zeijl MCT van den Eertwegh AJM. Switching to immune checkpoint inhibitors upon response to targeted therapy; the road to long-term survival in advanced melanoma patients with highly elevated serum LDH? Cancers. (2019) 11:1940. 10.3390/cancers11121940 31817189 PMC6966631

[127] Weber JS Sznol M Sullivan RJ Blackmon S Boland G Kluger HM. A serum protein signature associated with outcome after anti-PD-1 therapy in metastatic melanoma. Cancer Immunol Res. (2018) 6:79–86. 10.1158/2326-6066.CIR-17-0412 29208646

[128] Luo P Wu S Yu Y Ming X Li S Zuo X. Current status perspective biomarkers in AFP negative HCC: towards screening for diagnosing hepatocellular carcinoma at an earlier stage. Pathol Oncol Res. (2020) 26:599–603. 10.1007/s12253-019-00585-5 30661224

[129] Shao YY Lin ZZ Hsu C Shen YC Hsu CH Cheng AL. Early alpha-fetoprotein response predicts treatment efficacy of antiangiogenic systemic therapy in patients with advanced hepatocellular carcinoma. Cancer. (2010) 116:4590–6. 10.1002/cncr.25257 20572033

[130] Lee P.-C. Chao Y Chen M.-H. Lan K.-H. Lee C.-J. Lee IC. Predictors of response and survival in immune checkpoint inhibitor-treated unresectable hepatocellular carcinoma. Cancers. (2020) 12:182. 10.3390/cancers12010182 31940757 PMC7017111

[131] Dal Bello MG Filiberti RA Alama A Orengo AM Mussap M Coco S. The role of CEA, CYFRA21-1 and NSE in monitoring tumor response to Nivolumab in advanced non-small cell lung cancer (NSCLC) patients. J Transl Med. (2019) 17:74. 10.1186/s12967-019-1828-0 30849967 PMC6408784

[132] Goldberg SB Narayan A Kole AJ Decker RH Teysir J Carriero NJ. Early assessment of lung cancer immunotherapy response via circulating tumor DNA. Clin Cancer Res. (2018) 24:1872–80. 10.1158/1078-0432.CCR-17-1341 29330207 PMC5899677

[133] Cabel L Proudhon C Romano E Girard N Lantz O Stern M-H. Clinical potential of circulating tumour DNA in patients receiving anticancer immunotherapy. Nat Revi Clin Oncol. (2018) 15:639–50. 10.1038/s41571-018-0074-3 30050094

[134] Douillard J-Y Ostoros G Cobo M Ciuleanu T Cole R McWalter G. Gefitinib treatment in EGFR mutated caucasian NSCLC: circulating-free tumor DNA as a surrogate for determination of EGFR status. J Thor Oncol. (2014) 9:1345–53. 10.1097/JTO.0000000000000263 25122430 PMC4224589

[135] Vétizou M Pitt JM Daillère R Lepage P Waldschmitt N Flament C. Anticancer immunotherapy by CTLA-4 blockade relies on the gut microbiota. Science (New York, NY). (2015) 350:1079–84. 10.1126/science.aad1329 26541610 PMC4721659

[136] Sivan A Corrales L Hubert N Williams JB Aquino-Michaels K Earley ZM. Commensal Bifidobacterium promotes antitumor immunity and facilitates anti-PD-L1 efficacy. Science. (2015) 350:1084–9. 10.1126/science.aac4255 26541606 PMC4873287

[137] Zheng Y Wang T Tu X Huang Y Zhang H Tan D. Gut microbiome affects the response to anti-PD-1 immunotherapy in patients with hepatocellular carcinoma. J Immunother Cancer. (2019) 7:193. 10.1186/s40425-019-0650-9 31337439 PMC6651993

[138] Ren Z Li A Jiang J Zhou L Yu Z Lu H. *Gut* microbiome analysis as a tool towards targeted non-invasive biomarkers for early hepatocellular carcinoma. Gut. (2019) 68:1014–23. 10.1136/gutjnl-2017-315084 30045880 PMC6580753

